# A Qualitative Study Exploring Access to Mental Health and Substance Use Support among Individuals Experiencing Homelessness during COVID-19

**DOI:** 10.3390/ijerph19063459

**Published:** 2022-03-15

**Authors:** Emma A. Adams, Jeff Parker, Tony Jablonski, Joanne Kennedy, Fiona Tasker, Desmond Hunter, Katy Denham, Claire Smiles, Cassey Muir, Amy O’Donnell, Emily Widnall, Kate Dotsikas, Eileen Kaner, Sheena E. Ramsay

**Affiliations:** 1 Population Health Sciences Institute, Newcastle University, Newcastle upon Tyne NE3 4ES, UK; c.smiles2@newcastle.ac.uk (C.S.); c.muir@newcastle.ac.uk (C.M.); amy.odonnell@newcastle.ac.uk (A.O.); eileen.kaner@newcastle.ac.uk (E.K.); sheena.ramsay@newcastle.ac.uk (S.E.R.); 2 HeathNow, Crisis, City House 1 City Road, Newcastle upon Tyne NE1 2AF, UK; 3 Pathway, 4th Floor East, 250 Euston Road, London NW1 2PG, UK; 4 Crisis Pie Team, 66 Commercial Street, London E1 6LT, UK; 5 Expert by Experience Network, Fulfilling Lives Newcastle Gateshead, Gateshead NE8 4DY, UK; 6 Newcastle University Medical School, Newcastle University, Newcastle upon Tyne NE2 4HH, UK; k.denham@newcastle.ac.uk; 7 Population Health Sciences, Bristol Medical School, University of Bristol, Bristol BS8 2PS, UK; emily.widnall@bristol.ac.uk; 8 Division of Psychiatry, UCL, London W1T 7BN, UK; katharine.dotsikas.18@ucl.ac.uk

**Keywords:** homelessness, multiple complex needs, mental health, substance use, health care access, COVID-19, health inequalities

## Abstract

People experiencing homelessness have higher rates of mental ill-health and substance use and lower access to health services compared to the general population. The COVID-19 pandemic led to changes in service delivery across health and social care services, with many adopting virtual or telephone support for service users. This paper explores the experiences of access to community-based mental health and substance use support for people experiencing homelessness during the COVID-19 pandemic. Qualitative telephone interviews were conducted with 10 women and 16 men (ages 25 to 71) who self-identified as experiencing homelessness in North East England between February and May 2021. With five individuals with lived experience, results were analysed using inductive reflexive thematic analysis. Reactive changes to support provision often led to inadvertent exclusion. Barriers to access included: physical locations, repetition of recovery stories, individual readiness, and limited availability. Participants suggested creating services reflective of need and opportunities for choice and empowerment. Community mental health and substance use support for people experiencing homelessness should ensure the support is personalised, responsive to need, inclusive, and trauma-informed. The findings of this research have important implications for mental health and substance use policy and practice for individuals who experience homelessness during a public health crisis.

## 1. Introduction

The COVID-19 pandemic has amplified health inequalities and adversity among people who were already facing social and material disadvantage [1,2,3]. Indeed, historical evidence indicates that some groups are more susceptible to harm than others during pandemics and comparable crises [3,4,5], for example, individuals experiencing homelessness. However, fear of negative impacts from COVID-19 extend beyond having the infection to impacts on mental health (such as anxiety, isolation, and becoming mentally unwell, access to support) [6]. Early longitudinal evidence suggests that in the general population the prevalence of anxiety, depression, and other psychological impacts remained relatively unchanged [7,8]. For people experiencing homelessness, there is the potential that lockdowns and social distancing measures could negatively impact their mental health due to existing fears around forced incarceration and hospitalization [9]. The longer-term impacts to mental health from COVID-19 for this population remain unknown [10]. This is a particular concern since homelessness often co-exists with mental ill-health, substance use, and offending [11]. This co-occurrence of vulnerabilities is broadly defined as multiple complex needs, multiple exclusion homelessness, and severe and multiple disadvantage [12,13,14]. This co-occurrence and their interactions create a mutually reinforcing cycle, leading to further marginalisation and making it harder to access support [11,15,16,17,18].

People experiencing homelessness face disproportionately high rates of substance use and mental ill-health compared to the general population [11,19,20], yet have some of the lowest rates of engagement when it comes to accessing support [21,22,23]. Although provision is not always integrated, support for mental health and substance use crosses statutory health, social care, and voluntary sectors [24]. Examples of support may include, but is not limited to: specialist mental health services, assertive outreach, peer support, housing first, street support, assessment, trauma-informed care, psychologically informed environments, psychotherapy, street, support, and detox services [24,25,26,27]. Despite the variety of services, a survey found that essential mental health and addictions services are often unavailable in most areas and access is becoming increasingly difficult [25]. Epidemiological data has shown that people with co-occurring mental health and substance use (dual diagnosis) are less likely to access and use treatment [28,29,30]. It is likely that these rates would be even lower for homeless people, whose lack of a stable or permanent address can be a barrier to appointment-based care, where letters are sent to addresses or service provision is based on geographical regions [31].

Evidence demonstrating the disproportionate health and social care need and barriers to access people experiencing homelessness face is abundant [11,32,33,34,35]. An integrative literature review including qualitative and quantitative studies exploring access to treatment among individuals with dual diagnosis found two categories of barriers: personal characteristics and structural factors [36]. Personal characteristics barriers encompassed personal vulnerabilities (such as diagnosis-related symptoms associated with co-occurring substance use and mental ill health) and personal beliefs (such as lack of institutional trust, cultural beliefs, stigma) [37,38,39,40]. Whereas structural factors focus on availability, lack of appropriate diagnosis, and structural barriers to service provision (such as long waiting lists and gender-specific treatment options) [32,36,41,42]. Prior negative care experiences and lack of knowledge about the benefits of accessing health and social care support were continued barriers even after receiving housing accommodation [32]. However, most studies around barriers to accessing support for mental health and substance use by people experiencing homelessness have been quantitative. There remains a need for qualitative studies to better understand the experiences of homeless people on accessing support for mental ill-health and substance use.

Measures introduced to limit the spread of COVID-19 from March 2020 onwards led to substantial changes to daily life in the UK and elsewhere. Measures included social distancing, mandating people to ‘stay home’, and the closure of non-essential businesses and many face-to-face services. This meant many health and social care providers who traditionally offered support in-person transitioned to reduced capacity [43], socially distanced and remote support (such as telephone, Zoom, Skype, or Microsoft Teams) [43,44,45,46], and in some cases stopping services for periods of time [46]. The creative responses to offer support remotely demonstrated the ability of organisations to recover and adapt to the dynamic situation; effective adaption is essential for resilience with mental health and substance use services [47]. In cases where service provision was stopped, the organisation lacked resiliency and was ill-prepared/equipped for the changing circumstances.

For individuals experiencing homelessness, the initiatives introduced during COVID-19 focused on preventing disease transmission and providing temporary accommodation to house individuals sleeping rough [48]. Low substance use treatment rates coupled with high drug related deaths during COVID-19 suggests initiatives did not address the substance use needs of people experiencing homelessness [49]. This suggests that the health system was likely ill-prepared/equipped to meet these needs of people experiencing homelessness during the pandemic. Recent COVID-19 studies have highlighted a gap in understanding the depth of experiences for those who were dealing with mental health and substance use and particularly among people experiencing homelessness [45,46,49,50].

To better understand access to community-based mental health and substance use services, we conducted a qualitative study with people who experienced homelessness in North East England. We explored their experiences during the COVID-19 pandemic with accessing support, perceptions of barriers to access, and on what they might change to make support more accessible. Findings from this study will provide valuable information for building a more resilient healthcare system, which are better able to respond to current and future crises [47].

## 2. Materials and Methods

This qualitative study was informed by participatory action research approaches to ensure the study, findings, and recommendations were grounded in the experiences of those who are directly affected by the topic of the research [51,52,53,54]. Eligibility criteria were lived experience of homelessness, substance use, and/or mental ill-health. Initial interest was by male Experts by Experience, so the lead researcher made a specific request for females. After individuals expressed interest, E.A.A met with each person individually to clarify any concerns, explain expectations, and confirm participation. Experts by Experience then became part of the core project team and assisted in the design of the study, understanding of interviews, and sharing of findings. Approaches were based on best practices for community-based research with vulnerable populations [54]. To build rapport and reduce power imbalances between the lead researcher (E.A.A) and the Experts by Experience, Experts by Experience suggested the days and location (either Zoom or in-person) for meetings, refreshments (e.g., tea/coffee, cookies, and health snacks) were offered at all in-person meetings, and feedback was encouraged and listened to [55]. Using a cross-sectional design, semi-structured interviews were conducted over the phone between February and May 2021 during the COVID-19 pandemic.

### 2.1. Recruitment, Procedures, and Data Collection

Recruitment used purposive sampling through gatekeepers in housing and charity sectors alongside ‘Experts by Experience’ networks, followed by snowballing among participants. Individuals self-identified as experiencing homelessness during the COVID-19 pandemic, over 18 years of age, and wanting to access community mental health and addiction support within the region. Individuals did not need to have engaged with the support over the last year but did need to express an interest in seeking help. A broad definition of homelessness was used, including different types of homelessness, (e.g., rough sleeping, those in temporary accommodations such as hostels, those staying with friends or sofa surfing, and those who approach the local authority for housing) [56]. Given the frequently changing contexts and complex health and social care needs experienced by individuals within this population, efforts were made to accommodate re-scheduling interviews, calling different telephone numbers, and providing individuals with multiple opportunities to participate if they were initially unresponsive [57]. Thirty-one individuals expressed interest and a total of 26 participated; 2 could not be reached following multiple attempts to contact via hostels or referrer; 1 passed away before participating; 1 withdrew prior to data collection, and 1 moved on from their current hostel and did not have follow-up contact details. Participant recruitment continued until data sufficiency had been reached, no new themes, and anyone who had expressed interest previously was given an opportunity to participate.

Prior to participating, individuals were given an information sheet, assured anonymity and confidentiality, the option to withdraw at any time, an opportunity to ask any questions, and provided verbal or written consent. E.A.A conducted individual, telephone semi-structured interviews using a topic guide that covered the following areas: types of community-based mental health and addiction and interest in accessing them; experiences of access during the pandemic; and suggestions for improvement. The topic guide was developed with input from Experts by Experience and those who support people experiencing homelessness. Interviews ranged from 20 min in length to 80 min depending on what an individual was willing to share or their personal experiences. Individuals received a £30 voucher for a grocery store as a thank you for participating.

Ethics approval was granted by the Faculty of Medical Sciences Research Ethics Committee, part of Newcastle University’s Research Ethics Committee (ref: 2034/6698/2020.)

### 2.2. Analysis

Interviews were digitally recorded, transcribed, anonymised, and checked for accuracy. Analysis was informed by Braun and Clarke’s inductive reflexive thematic analysis [58]. The recursive process and nonlinear stages enabled shared meaning in the data focused on a central concept to be conceptualised as themes early in the analysis [59]. Led by the lead researcher (E.A.A), the analysis applied participatory approaches to work with five ‘Experts by Experience’ (J.P., T.J., J.K., F.T., D.H.) through a series of 10 Zoom and in-person workshops. We began by reading, listening, and re-reading transcripts followed by initial coding development for each transcript (all were done by E.A.A, and a portion were done by the five ‘Experts by Experience’). Preliminary themes were developed collectively based on initial coding and reviewed to determine patterns of shared meaning across transcripts. Naming and defining of themes and subthemes were refined with ‘Experts by Experience’ and discussed with all co-authors. The participatory approaches enhanced the quality of the research, as the ongoing reflective engagement led to richer and more nuanced understanding of the data and construction of themes [60]. All participants were given pseudonyms to maintain anonymity.

## 3. Results

Sixteen men and 10 women participated in the study, with ages ranging from 25 to 71 years (mean 40.7, standard deviation 11.4). All individuals self-identified as White British. No further demographic information was collected. Individuals reported a combination of mental health challenges (anxiety, depression, and other diagnosed conditions) and alcohol and drug use (cocaine, heroin, synthetic cannabinoids (spice), and marijuana) and diverse and multiple experiences of homelessness (ranging from sleeping on streets (rough sleeping) and staying with friends (couch surfing) to hostels and supported accommodation) during the COVID-19 pandemic. Table 1 presents an overview of the three main themes developed.

### 3.1. Inadvertent Exclusion

Participants reported that service provision had drastically changed over the course of the COVID-19 pandemic. Changes that were often made reactively to create support, led to inherent forms of exclusion.

#### 3.1.1. Mental Health Is Not 9-to-5

One of the most evident ways people felt overlooked was the fact that many providers only offered support between normal business hours; yet, people frequently felt that late at night was when they most needed support as ‘no one is awake’ and that was often when they hit ‘rock bottom’. Although this was not necessarily unique to COVID-19, individuals explained they wanted to be able to access some type of support during non-business hours; suggesting either a telephone line or texting service to ensure ‘somebody is there’ so you were never alone.

*“Aye, it should be open when you are feeling the worst. Sometimes when you are feeling your worst it is very late. Ya know, it’s dark, and it’s that’s when you feel your most loneliness. Like after 10 o’clock at night ya know, when there is no one around. People are in bed”.*

—*Keith, 45*

*“I want support 24/7”.*

—*Lily, 33*

Individuals reflected on a reduction in services during the pandemic and discussed how support they could normally access out-of-hours had been cut.

*“There’s an organisation [name removed] who I’ve sought help from in the past, but at this moment in time, they’re only open Monday to Friday, 10:00 a.m. to 4:00 p.m. If you’re having any episodes or if you need support outside of those hours, you can’t turn to them, where you used to be able to. They used to be open all sorts of hours and they even offered an emergency line for the weekends, but that’s all cut off now”.*

—*Mike, 33*

#### 3.1.2. Digital Exclusion

When speaking about experiences accessing services, individuals reflected on the pre-pandemic in-person support with general positivity although recognising it was not without its faults. Barriers included balancing the costs of travelling to appointments with having a meal or not feeling confident enough to meet people face-to-face. With social distancing restrictions coming into effect across England in response to COVID-19, there was a rapid transition to remote care provision to prevent services from closing their doors. Although this approach to care suited some people, others expressed frustration around feeling excluded. Exclusion took multiple forms including lack of device access and lack of digital literacy. Individuals expressed frustration around unspoken expectations that were required to access support.

*“He rang and went, “Right, if you join this Zoom link.” Obviously, I’m sat there as if to say, “Well what am I supposed to do? I haven’t got credit, I can’t ring up to say I can’t join the Zoom link.” Obviously, after I’d missed the Zoom link meeting, he rang me on the phone and went, “Why haven’t you joined?” I went, “Because I don’t have internet, I don’t have a laptop, I don’t have credit on my phone to get internet to do it.” He went, “Oh right, well we’ll do it over the phone now then.” It’s like, “Well why didn’t you ask about it?””.*

—*Danny, 29*

*“They expect you all to have these phones that do everything and we haven’t”.*

—*Sam, 37*

Across the interviews, conversations around not having access to Wi-Fi, laptops, or smart phones were plentiful. Where organisations provided devices for people experiencing homelessness, they spoke about issues of theft and the need for training to address gaps in digital literacy.

*“I dread to think how many phones I’ve had and they’ve been stolen or whatever. You couldn’t leave your phone lying anywhere. Everything goes. If you had a spit, it would be gone before it hit the floor”.*

—*Hannah, 43*

*“Computer illiterate. I’ve been on courses and that for it, but I get no further forward two weeks later from the first minute. I can’t log any technology in my head”.*

—*Alan, 54*

#### 3.1.3. Awareness of What Support Is out There

Knowledge of available care and support and who provides it was one of the most powerful barriers or facilitators to accessing support. Moreover, public spaces like libraries and coffee shops are typical locations to access information, however, COVID-19 lockdown restrictions had presented new barriers to accessing information from these locations. Participants suggested that information about support needed to be shared widely and not solely online, something that was heavily relied on during COVID-19.

*“Once you’re in that vicious circle, it can be very hard to access anything, you don’t know who to go to or who to speak to or who to contact, it’s just extremely difficult to know what to do”.*

—*Ian, 53*

*“Well, I think somebody coming out and actually talking to the girls, somebody being informed that there are services out there. The key is getting out into the hostels and knocking on the doors and saying, “This is what we’re offering, and would you like to be involved in it?” You’re not aware".*

—*Hannah, 43*

For those who accessed support, experiences depicted the variety of ways they found out about support (e.g., online, football match adverts, hostel workers, friends, bulletin boards, providers) and the fact that sometimes they were not looking for support when they found information about available support.

*“I just googled. Yeah it was um, I was googling how to kill myself and it came up with that. And I ended up using it. I’ve been using it for months”.*

—*Andrea, 25*

### 3.2. Barriers to Recovery

People shared negative experiences and barriers accessing support before and during COVID-19. These experiences shaped their attitudes towards accessing future support.

#### 3.2.1. Lack of Space for Recovery

With a push to house everyone sleeping rough during the pandemic, individuals reflected on their experiences of being provided with accommodation. Group accommodation was often provided to both current and ex-substance users. This group offering was often a negative experience and sometimes resulted in past users being targeted by drug dealers and facing behaviours they had moved on from. Participants also expressed frustration around experiencing this when accessing support in person.

*“They knew there was Spice use in this hostel. They shouldn’t have put me there. I was clean for seven months”.*

—*Sam, 37*

*“[treatment location] It’s not a nice place to go to when you’re trying to recover from drugs. They’re trying to sell you things outside, and inside the building to be honest. There’s always somebody trying to push something onto you. When you’re trying to recover yourself, it’s hard when people are putting things in your face”.*

—*Hannah, 43*

*“I don’t like going, but you got-you have to. Because, I’ve got away from it all, and yet you’re walking in and seeing people who are still using and they try and get you to buy some and things like that. [contrast to pandemic] While the pandemic is on, it’s all been changed to differently. You can’t go, they keep away and people get in touch with you over the phone. That would make it alright if it was always like that. That way you don’t have to bump into them”.*

—*Alan, 54*

These negative environments created further social isolation as individuals would try to isolate themselves from others to maintain their sobriety.

*“When I got put into shared accommodation it was quite hard because every one of the neighbours was on hard drugs like heroin and crack and stuff like that. Obviously, I’m no longer using. It’s like they try to pull you in, so I had to pretty much isolate myself when I was in there”.*

—*Danny, 29*

During COVID-19, support offered remotely often took place while individuals were in their shared accommodation. Individuals highlighted the importance of space and place during recovery and shared that their ideal location would be reflective of their current recovery stage, have ample space, and be supportive and welcoming.

#### 3.2.2. Disjointed Care and Repetition of Recovery Stories

Many people shared disconnected care experiences and discontinuity of care due to staff turnover, perhaps exacerbated by COVID-19 constraints. The constant need to repeat their stories caused many people to relive experiences.

*“I know. I am sick of getting new workers and having to explain again. Explaining me story to workers. You should get one worker. I’m sick. … I have done this for 17 years and I can’t do it anymore. I can’t”.*

—*Clare, 32*

*You know you get tired of times of saying—you just want to live a normal life again, you know. You explain your situation to one person, then you’ve, I’ve got to do it over and over and over again. It just seems like it’s never ending".*

—*Keith, 45*

One of the perceived solutions was creating an integrated system with increased collaboration and co-working across organisations. A few people shared experiences of how the pandemic led to providers working collaboratively to make sure people did not slip through the cracks.

*“Before lockdown, it was a bit of a jigsaw puzzle, everything was here, there and everywhere. Nobody was really communicating well enough together. But since lockdown, people have really honed in on their skills and they’ve had to learn to cope with different ways of doing things. I’ve got literally an appointment almost every day now, and I’ve got such a good routine going on, it’s amazing".*

—*Emily, 39*

#### 3.2.3. Not Ready for Recovery

Feeling ready to access support was perceived as extremely personal. Many people explained they were not ready to get help for their substance use or mental health as they had other priorities during COVID-19, including getting housed, a job, or their children back if they had been taken into state care. Individuals could not always articulate what could help them become ‘ready’.

*“I don’t know if the support is there but I’m not ready to ask for help”.*

—*Clare, 32*

*“When you’re feeling down like that and you’re wanting to go with stuff…”.*

—*Danny, 29*

Many felt they should be given adequate space and time and not be pressured into seeking help as it may lead to disengagement.

#### 3.2.4. Prioritization When Resources Are Scarce

A common thread across interviews was that people often met ‘brick walls’ when trying to access support. This felt even harder during the pandemic where services appeared fewer and farther between. Examples included waiting lists, requirements around referral pathways, not meeting eligibility criteria around ‘being sick enough,’ or being passed around between services.

*“I rung the Crisis team a lot. I was on the phone to them nine times in one night before they actually came out. […later explains] the Crisis team is pretty much the wrong name for them I would say”.*

—*Mike, 33*

A shared experience across interviews was the fact that accessing support was often challenging.

*“If you’re determined to climb to the top of that mountain, you’re going to get there. But it’s really, really difficult. So, you just have to shout and scream and try as hard as you can”.*

—*Emily, 39*

Experiences of being ignored or frequently passed from one place to another led to people feeling reluctant to access support.

*“They say “ah we can do this, we can do that” but it never seems to come off, or the supports not really that good or it’s bad for you, it’s not good for you. Most of them just leave you to do what you are doing. … Promises, false promises and it doesn’t happen”.*

—*Tommy, 38*

### 3.3. Building a System Responsive to Needs

Across the interviews, participants highlighted that before and during COVID-19, support was frequently offered using a structured approach that was disempowering for people experiencing homelessness.

#### 3.3.1. Disconnect between Service Provision and Needs

Amplified by frustration around the lack of support during COVID-19, people often felt services did not understand their needs. This meant not recognising or understanding the complex interaction of mental health and substance use. One suggestion was to include peer-led or peer supported approaches within services. This was a perceived opportunity to leverage lived experience knowledge to design services to reflect the complex needs faced by people experiencing homelessness.

*“Because they say, “Oh, you have to reduce your drinking,” but I can’t. I’ve got deep psychological issues, I can’t- So, I need the therapy in line with the reduction of alcohol. I need them in conjunction, that’s my biggest hurdle at the minute. It’s a vicious cycle, isn’t it?”.*

—*Emily, 39*

*“…it would have helped because we’d all be in the same boat anyway, so we could help each other with our own experiences. [later goes on to say] Yes, it would be somebody on your own level that has actually been through alcoholism or drug use”.*

—*Glen, 62*

Individuals reflected on how the complexity of co-occurring mental health and substance use meant there was uncertainty over when they would need help with mental health or substance use. This was a particular concern during COVID-19 where lives felt dominated by uncertainty around when things would return to ‘normal’. The best experiences of access were when individuals felt that providers recognised this uncertainty and supported individuals with constant reviews and check-ins.

*“Just because I’m feeling good this month, it doesn’t mean that in a month or two’s time, I am still feeling great. There are constant reviews and chats and contacts and stuff, which is great”.*

—*Liam, 26*

#### 3.3.2. Choice and an Active Voice

During COVID-19 people felt they lost control of their circumstances and were frustrated with being offered limited and no flexibility in options. One of the easiest ways to create a more positive experience of access was through giving individuals choice in their care. In the context of COVID-19, choice was offered by providing support through a variety of mediums; for example, face-to-face, online, in groups, or one-to-one.

*“Everyone’s different. So, a lot of people loved the Zoom, a lot of people like going to different meetings, a lot of people need a lot more support than other people. So, it’s about tailoring it or catering for the individual isn’t it, I suppose”.*

—*Andy, 46*

One of the key messages shared during interviews was that individuals need to be asked what they want, to be involved in decision making, and to feel empowered to be active participants in their care.

*“Like when people are saying, like they don’t want to be here, or anything in the mental health bag—don’t just hoy a prescription in their face. Like sit down with them and look at what they need".*

—*Carlie, 25*

*“Ask the person, ask the individual how they want to be helped, that’s the way forward for them, what help do they need”.*

—*Darren, 36*

## 4. Discussion

The urgent response to the global COVID-19 pandemic has led to new ways of working, which presented an opportunity to change how mental health and substance use support is provided. This study explored the experiences of access to community-based mental health and substance use support in people experiencing homelessness during the pandemic. The experiences of access shared took place while there were constraints to the mental health and substance use system (such as reduced service provision and staffing) brought about by the pandemic and pre-existing austerity. Although many experiences of access were negative, individuals did share some positive experiences or suggestions for service improvement.

Although this study focused on experiences during the pandemic, our findings illustrated some known barriers to accessing mental health and substance use support that existed prior to the pandemic and align with pre-pandemic research findings. These included services being restricted to business hours, repeating recovery stories, and limited support [61,62,63,64,65], suggesting an opportunity to improve service provision irrespective of times of austerity and crisis. Negative experiences and barriers to access highlight the need for a resilient mental health and substance use system that is responsive to the needs and circumstances of vulnerable groups such as people experiencing homelessness.

Experiences of exclusion took many forms across interviews. Many people felt they could not access mental health support when they needed it most (e.g., in the evening) or in the most suitable form for their needs or abilities. This echoes research prior to COVID-19 [61,62]. During conversations about remote out-of-hours support many discussed access issues surrounding not having smartphones, internet, or feeling uncomfortable accessing support online or transitioning to online support. Remote solutions therefore need to consider these barriers to ensure appropriate and inclusive access [46], but this also suggests that more creative out-of-hours provision solutions are needed.

Our findings emphasise that when additional needs (such as past negative experiences, dual diagnosis, material disadvantage) are not recognised, people experiencing homelessness are often unable to appropriately access support. Prior research has extensively explored the complex structural and individual circumstances surrounding people experiencing homelessness [33,34,61,66]. Our findings suggest that the instability around circumstances and needs requires recognition from service providers during times of heightened adversity. Participants’ suggestions of hiring and training people with lived experience could be one way to better target provision; existing evidence demonstrates improved treatment outcomes in mental health and substance use support when lived experience is integrated within care provision [27,67,68].

One of the most crucial messages within interviews was the need to have a choice in one’s care, particularly during the pandemic where people felt their loss of autonomy and control had worsened. The notion of rebuilding control has previously been highlighted within trauma-informed care approaches for homelessness [69]. In line with autonomy and control, our findings highlighted that not everyone is ready to access support but that continuing to increase awareness and knowledge of current support would equip individuals with the necessary information to access support at the right time for them. This knowledge is essential considering knowing where to go to access support is a central issue for people experiencing homelessness [70,71].

Negative environments for accessing support (such as high presence of drugs, lack of safety) were a source of frustration and led to subsequent care avoidance; a consequence seen in other studies [23,72]. Telephone support to accommodate COVID-19 restrictions meant some people finally felt comfortable accessing support as the environment changed. Like existing mental health and homeless literature [63,64,65], participants shared frustration around repeating their stories; likely a consequence of a siloed system of care/support. In a few cases, individuals shared how the pandemic was the first time they found services working collaboratively to support them—echoing other emerging evidence [66]. Lessons from providers on mechanisms for coordination across the system during COVID-19, could allow future approaches to be more bespoke and coordinated to ensure that the specific needs of people experiencing homelessness are addressed [73]. Finally, our findings highlight that many struggled to access support, particularly with the reduced service provision and increased waiting lists during COVID-19. Recognising that reduced service provision was perhaps unavoidable given the context, it is important to consider the impact these negative experiences have on future access and experiences.

### 4.1. Strengths and Limitations

A key strength to this study is its application of participatory approaches, which meaningfully brings the voice of people with lived experience to the study design, methodology and interpretation of findings. Involving individuals with lived experience throughout each phase of the data analysis meant that some of the initial codes were created based on their suggestions and theme refinement was influenced by their rich insights. Lived experience involvement also led to an action-oriented interpretation with a desire to improve future experiences of access, provision, and policy around community mental health and substance use support.

The study is limited by its localised recruitment in Newcastle and Gateshead; however, preliminary conversations with providers in other regions suggest the findings echo their experiences. Despite active recruitment effort, we were unable to recruit anyone who did not self-identify as White British, however, the proportion of individuals in North East England who do not identify as White British is less than 8% in the general population [74]. Discussions with local providers emphasised that contrary to other regions in England, most individuals who experience homelessness in North East England self-identify as White British. The study employed solely remote approaches to data collection due to the COVID-19 restrictions. Although research has shown the acceptability of remote data collection [75], the potential of sampling bias remains due to not reaching out to those without telephone or internet access or potentially those sleeping rough [76]. We attempted to mitigate this bias through working closely with hostels to ensure individuals had access to communal phones.

### 4.2. Implications for Practice and Research

Our findings suggest several actionable solutions for policymakers and providers in health, social care, and housing that are transferable outside the context of pandemics and times of crisis. These include providing choice (e.g., offer someone a choice between service provision online or in-person or offering choices for the time of day for provision), lived experience representation within support, making information about services accessible (e.g., communicating using simple and clear language [23]), ensuring appropriate and inclusive digital solutions (e.g., working with individuals with lived experience to co-create solutions or providing smartphones with internet access [46]), creating a safe space for accessing support (e.g., a service that feels safe, private, and is not overly crowded [23]), and creating bespoke and integrated care pathways. Research is needed to investigate the difference and factors associated with digital confidence and literacy among people experiencing homelessness. In all cases, solutions should be designed, implemented, and delivered with individuals with lived experience to ensure it is acceptable and appropriate for this population. Ensuring that provision is responsive and appropriate to those most in need is vital for integrated care systems across England.

Through working with individuals with lived experience, we were able to better understand the experiences of participants while being open to seeing solutions to challenges. Further research should look to understand the perspectives of those providing support and co-developing solutions that are equally acceptable to providers and those who experience homelessness.

## 5. Conclusions

COVID-19 presented an opportunity to learn from changes in service provision and has drawn attention to the need for more accessible and bespoke support for people experiencing homelessness. This study provided rich accounts on the (in)accessibility of mental health and substance use support, which have implications for developing future policy and practice responses. These implications are potentially beneficial for post-pandemic efforts to improve access to and increase the resilience of mental health and substance use support for people experiencing homelessness.

## Figures and Tables

**Table 1:** Themes and subthemes related to access to community mental health and substance use support for people experiencing homelessness during the COVID-19 pandemic.

Themes	Inadvertent Exclusion	Barriers to Recovery	Building a System Responsive to Needs
Subthemes	Mental health is not 9-to-5 Digital exclusion Awareness of what support is out there	Lack of space for recovery Disjointed care and repetition of recovery stories Not ready for recovery Prioritisation when resources are scarce	Disconnect between service provision and needs Choice and an active voice

## Data Availability

Summaries of the data presented in the study are available on request from the corresponding author. The data are not publicly available due to containing potentially identifiable information about participants.
